# Development and Validation of a TNF Family-Based Signature for Predicting Prognosis, Tumor Immune Characteristics, and Immunotherapy Response in Colorectal Cancer Patients

**DOI:** 10.1155/2021/6439975

**Published:** 2021-09-09

**Authors:** Zheng Xiao, Kechao Nie, Tong Han, Lin Cheng, Zheyu Zhang, Weijun Peng, Dazun Shi

**Affiliations:** ^1^Department of Integrated Traditional Chinese & Western Medicine, The Second Xiangya Hospital, Central South University, Changsha, Hunan 410011, China; ^2^Department of Nephrology, The Second Xiangya Hospital, Central South University, Changsha, Hunan 410011, China; ^3^Department of General Surgery, The Second Xiangya Hospital, Central South University, No. 139 Middle Renmin Road, Changsha, Hunan 410011, China; ^4^Department of Gynecology and Obstetrics, Xiangya Hospital, Central South University, Changsha, Hunan 410008, China

## Abstract

In this study, a comprehensive analysis of TNF family members in colorectal cancer (CRC) was conducted and a TNF family-based signature (TFS) was generated to predict prognosis and immunotherapy response. Using the expression data of 516 CRC patients from The Cancer Genome Atlas (TCGA) database, TNF family members were screened to construct a TFS by using the univariate Cox proportional hazards regression and the least absolute shrinkage and selection operator- (LASSO-) Cox proportional hazards regression method. The TFS was then validated in a meta-Gene Expression Omnibus (GEO) cohort (*n* = 1162) from the GEO database. Additionally, the tumor immune characteristics and predicted responses to immune checkpoint blockade in TFS-based risk subgroups were analyzed. Eight genes (TNFRSF11A, TNFRSF10C, TNFRSF10B, TNFSF11, TNFRSF25, TNFRSF19, LTBR, and NGFR) were used to construct the TFS. Compared to the high-risk patients, the low-risk patients had better overall survival, which was verified by the GEO data. In addition, a high TFS risk score was associated with high infiltration of regulatory T cells (Tregs), nonactivated macrophages (M0), natural killer cells, immune escape phenotypes, poor immunotherapy response, and tumorigenic and metastasis-related pathways. Conversely, a low TFS risk score was related to high infiltration of resting CD4 memory T cells and resting dendritic cells, few immune escape phenotypes, and high sensitivity to immunotherapy. Thus, the eight gene-based TFS is a promising index to predict the prognosis, immune characteristics, and immunotherapy response in CRC, and our results also provide new understanding of the role of the TNF family members in the prognosis and treatment of CRC.

## 1. Introduction

Colorectal cancer (CRC) is the fourth leading cause of cancer-related mortality worldwide, with a high incidence rate [1]. Although great improvements have been made in the diagnosis and treatment of CRC, the prognosis of CRC patients remains disappointing. Radical resection is the gold standard treatment for CRC patients. However, the recurrence rate of CRC within 2 years after undergoing radical resection has been reported to be high (nearly 50%), and half of the relapses were fatal [2]. Immunotherapy, especially inhibitors targeting immune checkpoints, including cytotoxic T-lymphocyte antigen-4 (CTLA4), programmed cell death protein 1 (PD-1), and programmed cell death 1 ligand 1 (PD-L1), has provided promising new approaches to improve the overall survival (OS) of patients with CRC [3–6]. In particular, recent studies have demonstrated that pembrolizumab (an anti-PD-1 monoclonal antibody) had improved efficacy and long-term clinical benefit for the subgroup of patients with microsatellite instability-high (MSI-H)/DNA mismatch repair-deficient (dMMR) CRC [7, 8]. However, as these immune checkpoint inhibitors have only exhibited clinical success in a small proportion of CRC patients [9], finding other immune checkpoint targets has important clinical implications.

Recently, increasing evidence has indicated that therapies related to costimulation of T cell responses by tumor necrosis factor (TNF) family members may represent another therapeutic approach, in addition to blocking the abovementioned immune checkpoints [10]. The TNF and TNF receptor (TNFR) superfamilies (TNFSF/TNFRSF) are composed of 19 ligands and 29 receptors. The communication pathways mediated by TNFSF/TNFRSF members orchestrate inflammation and control cell survival, proliferation, and differentiation [11]. TNFSF/TNFRSF members exhibit proinflammatory properties by activating the nuclear factor- (NF-) *κ*B pathway, a central pathway in the processes to combat both pathogens and cancers [12]. Inflammation also enhances tumor proliferation, metastasis, and angiogenesis in many types of cancer [13]. Anti-TNF therapy has been reported to be associated with a decreased risk of CRC in inflammatory bowel disease [14]. Therefore, investigation of the control of TNFSF/TNFRSF activities may provide new insights into treating cancer. In fact, therapeutic approaches that target TNFSF/TNFRSF members (including GITR, CD30, CD40, and OX40) are currently being studied in preclinical or clinical trials for the treatment of various cancers, including CRC [15–19]. However, the expression profiles and clinical significance of these members in CRC remain unclear.

In this study, the expression profiles and clinical significance of TNF family members in 516 CRC cases from The Cancer Genome Atlas (TCGA) database were explored. An eight-gene prognostic signature based on the TNF family members, designated the TNF family-based prognostic signature (TFS), was constructed using the univariate Cox proportional hazards regression and the least absolute shrinkage and selection operator- (LASSO-) Cox proportional hazards regression method. It was validated using Gene Expression Omnibus (GEO) datasets. Additionally, the proportions of tumor-infiltrating immune cells and immunotherapy responses were compared between the TFS-based high- and low-risk groups.

## 2. Materials and Methods

### 2.1. Datasets

We collected gene expression, clinical, overall survival (OS) rate, and somatic mutation data on 516 CRC patients from the TCGA database (https://portal.gdc.cancer.gov/repository), which was used as the training set. The validation sets were four large GEO datasets (http://www.ncbi.nlm.nih.gov/geo) containing gene expression, clinical, and OS data from CRC patients, comprising 177 cases (GSE17536), 55 cases (GSE17537), 579 cases (GSE39582), and 351 cases (GSE87211).

### 2.2. TFS Construction and Validation

The univariate Cox regression analysis was used to identify the TNF family genes that have predictive ability in CRC. Genes with *P* < 0.1 were then subjected to a LASSO-Cox regression analysis to identify the most valuable prognostic genes. Finally, a TFS was established in the 516 patients with CRC in the TCGA cohort. Each CRC patient's TFS risk score was calculated based on the coefficients from the LASSO regression analysis and their corresponding gene expression data. The optimal cutoff for dividing the CRC patients into low- and high-risk groups was determined using the “survival” package in R. Finally, the Kaplan–Meier survival curve analysis with the log-rank test was conducted to evaluate the prognostic value of the TFS.

To comprehensively evaluate the prognostic value of the TFS in an external cohort, we evaluated the prognostic value in a meta-GEO cohort (based on integrating all four GEO datasets), which involved 1162 CRC cases with survival data, and in the four GEO datasets individually.

### 2.3. Gene Ontology (GO) and Kyoto Encyclopedia of Genes and Genomes (KEGG) Pathway Enrichment Analyses

The differentially expressed genes (DEGs) between the high- and low-risk patients were identified based on the following criteria: absolute value of log2 (fold change (FC)) ≥ 1 and false discovery rate (FDR) < 0.05. The main GO terms (biological processes, molecular functions, and cell components) and KEGG pathways associated with the DEGs were evaluated using GO and KEGG pathway enrichment analyses. The analyses were performed using the “clusterProfiler” package in R software.

### 2.4. Immune Cell Infiltration and the Tumor Microenvironment (TME) in the TFS-Based Risk Subgroups

Estimation of Stromal and Immune cells in Malignant Tumors using Expression data (ESTIMATE) was used to compute the tumor purity, stromal score, immune score, and ESTIMATE score for the CRC cases. CIBERSORT analysis (involving the LM22 immune cell gene signature file) was used to assess the proportions of 22 tumor-infiltrating immune cell types in patients with CRC in the TCGA cohort. CIBERSORT is a novel tool that quantifies the abundance of 22 immune cells in a complex tissue according to gene expression profiles [20].

### 2.5. Tumor Immune Dysfunction and Exclusion (TIDE) Analysis

The TIDE algorithm was used to predict clinical responses to immune checkpoint blockade (ICB). Patients with low TIDE prediction scores tend to have a good response to immunotherapy, while patients with a high TIDE prediction score are predicted to be nonresponders [21].

### 2.6. TFS-Based Nomogram for Predicting OS

Traditional clinically important factors and the TFS were subjected to univariate regression analysis to select the factors with significant prognostic value. The significant factors were then used in a multivariate regression analysis. Finally, a nomogram was constructed based on the independent prognostic factors in the multivariate analysis. Calibration curves were used to evaluate whether the nomogram-predicted survival was consistent with the actual survival.

### 2.7. Statistical Analysis

The Kaplan–Meier method was used to assess the OS in the high- and low-risk groups, with a log-rank test being used to assess the significance of the difference in OS between the two groups. Independent prognostic factors were determined by the Cox proportional hazards regression. *P* < 0.05 indicated significance in all statistical analyses.

## 3. Results

### 3.1. Identification of the Prognostic TNF Family Genes in CRC

The 47 well-defined TNF family genes were evaluated, comprising 18 TNFSF members and 29 TNFRSF members. First, a univariate Cox proportional hazards regression analysis was performed on the TNF family gene expression data in the 516 CRC cases in the TCGA cohort. The clinical characteristics of these CRC patients are listed in Table 1. Nine genes were identified as being significantly associated with OS (*P* < 0.1) (Table 2). Among these nine genes, four (TNFRSF25, TNFRSF19, LTBR, and NGFR) with a hazard ratio > 1 were identified as risk factors, while the other five (TNFRSF11A, TNFRSF10C, TNFRSF10B, TNFSF11, and FAS) with a hazard ratio < 1 were identified as protective factors.

### 3.2. Establishing a TFS Using the CRC Patients in the TCGA Cohort

**Figure 1:**
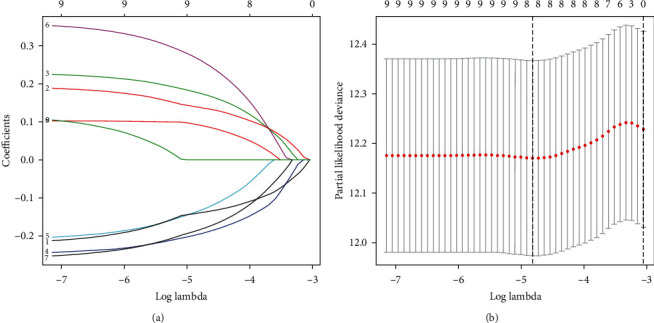
Tenfold cross-validation for tuning parameter selection. (a) LASSO coefficient profiles of the nine prognostic genes (originally identified in the univariate Cox regression analyses). (b) Plots of the cross-validation error rates.

**Figure 2:**
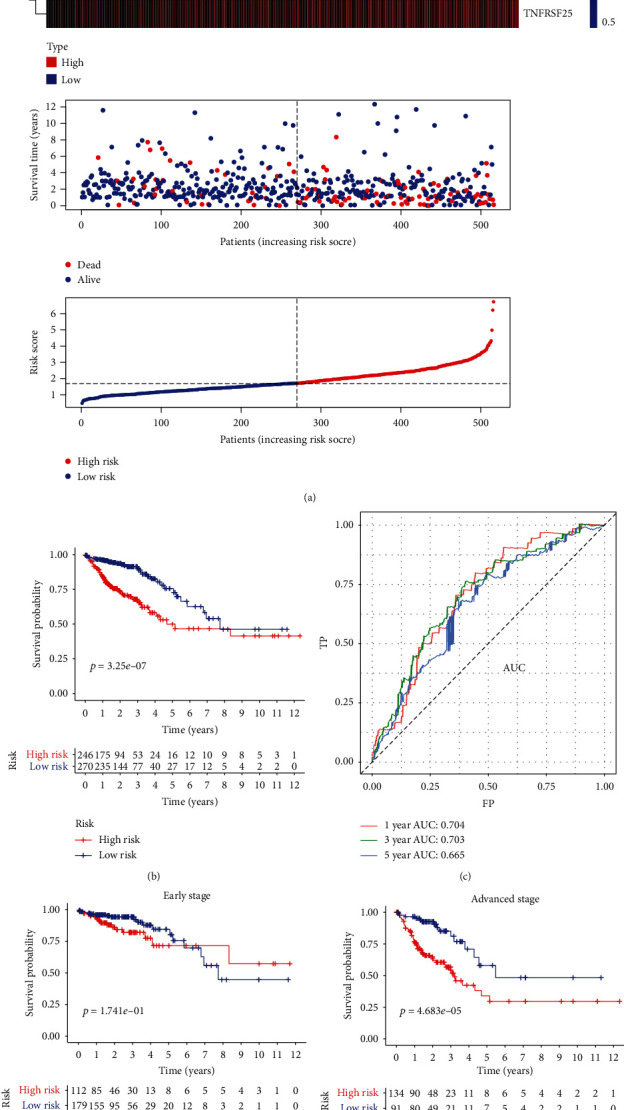
Establishment of the TNF family-based signature (TFS). (a) Heatmap of the mRNA expression of the eight genes in the TFS, survival status, and TFS risk scores in the high- and low-risk groups. (b) The Kaplan–Meier curves of the OS of CRC patients in the high- and low-risk groups. (c) Validation of the prognostic value of the eight-gene TFS at 1, 3, and 5 years via time-dependent ROC curve analysis in the TCGA cohort. The Kaplan–Meier survival analyses of the patients in the high- and low-risk groups with (d) early-stage CRC (stages I and II) (*n* = 291) and (e) advanced-stage CRC (stages III and IV) (*n* = 225).

To build the optimum TFS (risk model), the nine genes were subjected to a LASSO-Cox regression analysis (Figure 1), which led to the establishment of the following formula involving eight TNF family members: TFS risk score = (−0.1344 × TNFRSF10B expression) + (0.2647 × LTBR expression) + (−0.1953 × TNFRSF10C expression) + (0.1843 × TNFSF11 expression) + (0.1764 × TNFRSF19 expression) + (0.0916 × NGFR expression) + (−0.1395 × TNFRSF11A expression) + (0.1387 × TNFRSF25 expression) (Figure 1). Thereafter, the TFS risk score of each patient was calculated based on the above formula. The optimal cutoff (1.72) was computed and subsequently used to divide the patients into high-risk (*n* = 246) and low-risk (*n* = 270) subgroups. The gene expression profiles, TFS risk scores, and survival status of the CRC patients in the TCGA cohort are displayed in Figure 2(a).

The Kaplan–Meier survival analyses showed that the OS was lower in the high-risk group (Figure 2(b)*P* < 0.00001) than in the low-risk group. Next, the time-dependent area under the receiver operating characteristic (AUC-ROC) curve values were calculated to evaluate the ability of the TFS to predict 1-, 3-, and 5-year OS in the TCGA cohort. These values were 0.704, 0.703, and 0.665, respectively (Figure 2(c)). As the treatment strategies and prognosis are remarkably different between early-stage (stages I and II) and advanced-stage (stages III and IV) CRC, the TFS was further applied to these different clinical stages. The advanced-stage patients had worse OS in the high-risk group than in the low-risk group (Figure 2(e)) (log-rank test, *P* < 0.0001).

### 3.3. Verification of the TFS in Four Independent Cohorts and the Meta-GEO Cohort

**Figure 3:**
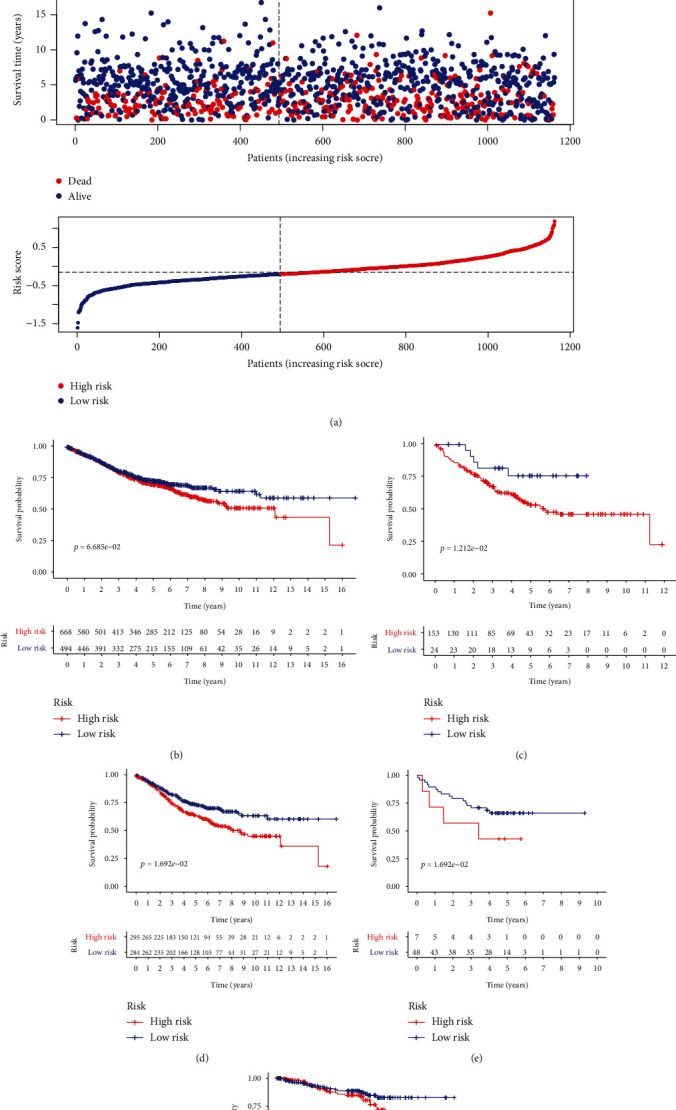
Validation of the eight-gene TNF family-based signature (TFS) for CRC patients using GEO data. (a) Heatmap of the mRNA expression of the eight genes in the TFS, survival status, and TFS risk scores in the high- and low-risk groups. The Kaplan–Meier survival analyses of the patients in the high- and low-risk groups in the (b) meta-GEO cohort (*n* = 1162), and in the (c) GSE17536 (*n* = 177), (d) GSE39582 (*n* = 579), (e) GSE17537 (*n* = 55), and (f) GSE87211 (*n* = 351) datasets.

To evaluate the prognostic robustness of the eight-gene TFS, its performance in the four independent GEO datasets (GSE17536, GSE39582, GSE17537, and GSE87211) and the meta-GEO cohort was further assessed using the same risk formula. Based on the optimal TFS risk score cutoff, patients in the validation cohorts were divided into high- and low-risk groups. The mRNA expression of the eight genes in the TFS, survival status, and TFS risk score in the meta-GEO cohort are presented in Figure 3(a). As expected, the Kaplan–Meier results showed that the high-risk patients had worse OS compared to the low-risk group, in the GSE17536 dataset (cutoff = −0.43; log-rank test, *P* = 0.0121) (Figure 3(c)), in the GSE39582 dataset (cutoff = −0.2; log-rank test, *P* = 0.2372) (Figure 3(d)), in the GSE17537 dataset (cutoff = 0; log-rank test, *P* = 0.0169) (Figure 3(e)), and in the GSE87211 dataset (cutoff = −0.02; log-rank test, *P* = 0.3026) (Figure 3(f)). As *P* > 0.05 for two of the above four independent datasets, the prognostic value of the TFS was further evaluated in the meta-GEO CRC cohort. The results revealed that the TFS had high predictive ability for patients with CRC (cutoff = −0.2; log-rank test, *P* < 0.001) (Figure 3(b)).

### 3.4. Assessment of Immune Cell Infiltration and the TME in the High- and Low-Risk Groups

**Figure 4:**
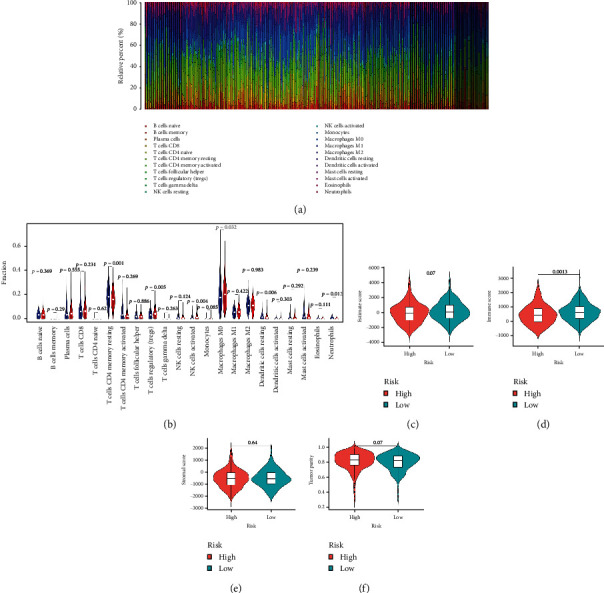
Tumor-infiltrating immune cells and tumor microenvironment (TME) in high- and low-risk CRC patients. (a) Distribution of 22 tumor-infiltrating immune cells in 516 CRC patients. (b) Comparison of the proportions of tumor-infiltrating immune cells between the high- and low-risk groups. (c–f) Associations of the TFS risk score with the ESTIMATE score, immune score, stromal score, and tumor purity in CRC patients in the TCGA cohort.

To assess the immune cell infiltration associated with the two TFS risk subgroups, CIBERSORT with LM22 was used for each CRC case in the TCGA cohort to evaluate the proportions of 22 immune cells (Figure 4(a)). There was high infiltration of resting CD4 memory T cells and resting dendritic cells in the low-risk group, while there was high infiltration of regulatory T cells (Tregs), nonactivated macrophages (M0), activated natural killer (NK) cells, and neutrophils in the high-risk group (*P* < 0.001) (Figure 4(b)). Additionally, the ESTIMATE algorithm was employed to evaluate the differences in ESTIMATE score, stromal score, immune score, and tumor purity in patients with CRC between the low- and high-risk groups. We found that the TFS was negatively associated with the ESTIMATE score and immune score, while the TFS was positively associated with stromal score and tumor purity. However, only the negative association between TFS and immune score was significant (*P* < 0.05) (Figures 4(c)–4(f)).

### 3.5. Associations between the TFS and Immunotherapy Response

As the TFS successfully predicted the OS rate of CRC patients, we further investigated its predictive ability regarding immunotherapy responses.

**Figure 5:**
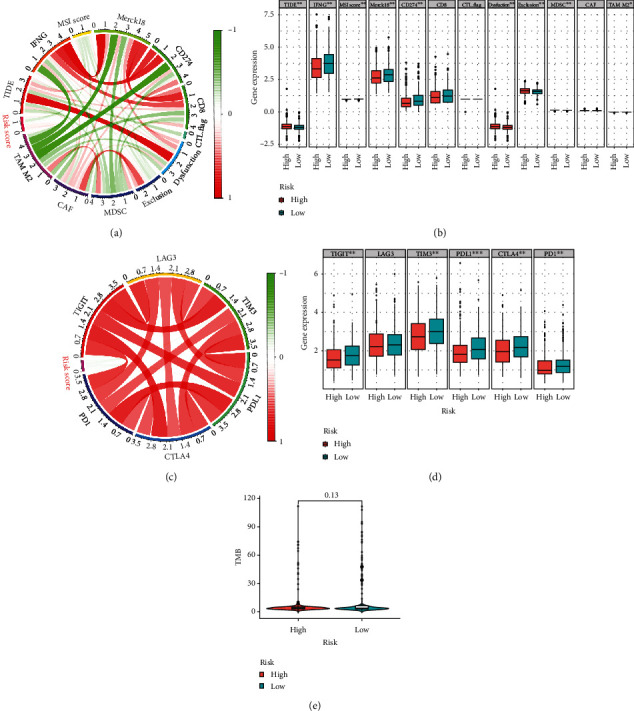
Comparison of the expression of immunotherapy response biomarkers in the high- and low-risk groups. (a) Comparisons of Tumor Immune Dysfunction and Exclusion (TIDE) Score, interferon-gamma (IFNG), microsatellite instability (MSI) Score, Merck18, CD274, CD8, CTL.flag (an indicator of the expression of five cytotoxic T-lymphocyte markers), Dysfunction (T cell dysfunction), Exclusion (T cell exclusion), myeloid-derived suppressor cells (MDSC), cancer-associated fibroblasts (CAF), and tumor-associated macrophages (TAM) M2 in the high- and low-risk groups. (b) Comparisons of mRNA expression of immune checkpoint molecules (PD-L1, PD-1, CTLA3, TIM-3, LAG-3, and TIGIT) in the high- and low-risk groups.

First, a correlation analysis between the TFS and immune checkpoint proteins, including CTLA4, PD-1, PD-L1, lymphocyte activation gene-3 (LAG-3), T cell immunoglobulin and immunoreceptor tyrosine-based inhibition motif domain (TIGIT), and T cell immunoglobulin-3 (TIM-3), in the 516 CRC patients in the TCGA dataset was performed. There were negative associations between the TFS and these immune checkpoint proteins (*P* < 0.01), except for LAG-3, which was not associated with the TFS (*P* > 0.05) (Figures 5(c) and 5(d)).

Thereafter, the TIDE algorithm was applied to predict responses to ICB. Interestingly, there were significantly higher values in the high-risk group regarding the TIDE Score, T cell dysfunction (Dysfunction), T cell exclusion (Exclusion), and myeloid-derived suppressor cells (MDSC). In contrast, there were significantly lower values in the high-risk group regarding interferon-gamma (IFNG), MSI score, Merck18, CD274, and tumor-associated macrophage M2 (Mann–Whitney *U* test *P* < 0.001) (Figures 5(a) and 5(b)). Finally, we analyzed the difference in the tumor mutation burden (TMB) between high- and low-risk patients, and no significant difference was found (Figure 5(e)).

### 3.6. Functional Enrichment Analyses of the DEGs between the High- and Low-Risk Groups

**Figure 6:**
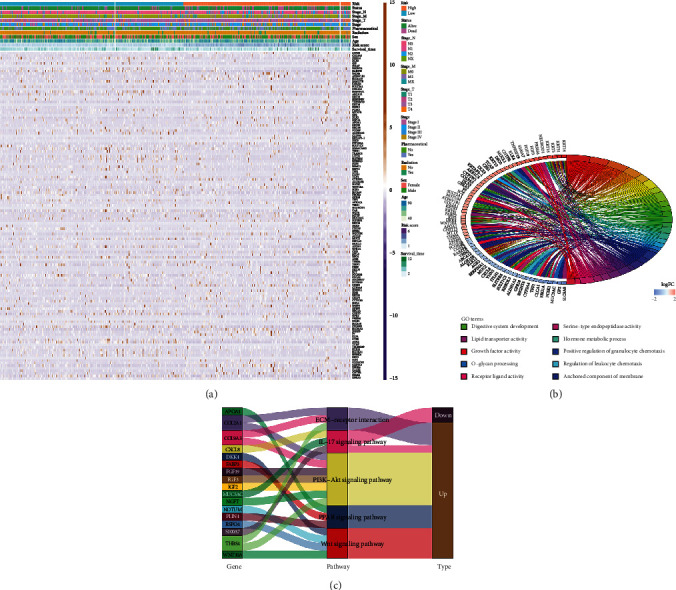
Enrichment analyses of differentially expressed genes (DEGs) between the high- and low-risk groups. (a) Heatmap of DEGs in patients in the high- and low-risk groups based on TNF family-based signature (TFS) risk scores. (b) Gene Ontology (GO) terms and (c) Kyoto Encyclopedia of Genes and Genomes (KEGG) pathways associated with the DEGs.

Based on the criteria of absolute value of log2(fold change (FC)) ≥ 1 and FDR < 0.05, 127 DEGs were identified between the high- and low-risk groups. Among them, 74 genes were upregulated (log2FC > 1) and 53 genes were downregulated (log2FC < 1) in the high-risk group. Subsequently, the 127 DEGs were subjected to GO and KEGG analyses to further understand the biological functions and signaling pathways related to these genes. The GO analysis indicated that the DEGs were mainly enriched in the following GO biological processes: digestive system development, O-glycan processing, hormone metabolic process, and regulation of leukocyte chemotaxis. The GO molecular functions of the DEGs included lipid transporter activity, serine-type endopeptidase activity, receptor ligand activity, positive regulation of granulocyte chemotaxis, and growth factor activity. The main enriched GO cell component was the anchored component of membrane (Figure 6(b)). KEGG pathway analysis indicated that the DEGs were highly associated with the peroxisome proliferator-activated receptor (PPAR), phosphoinositide 3-kinase (PI3K-) Akt, interleukin- (IL-) 17, and Wnt signaling pathways and extracellular matrix- (ECM-) receptor interaction, which have been confirmed to be involved in cancer initiation and progression (Figure 6(c)) [22–26]. These biological functions and pathways may contribute to the roles of DEGs in the development of CRC.

### 3.7. TFS-Based Nomogram

**Figure 7:**
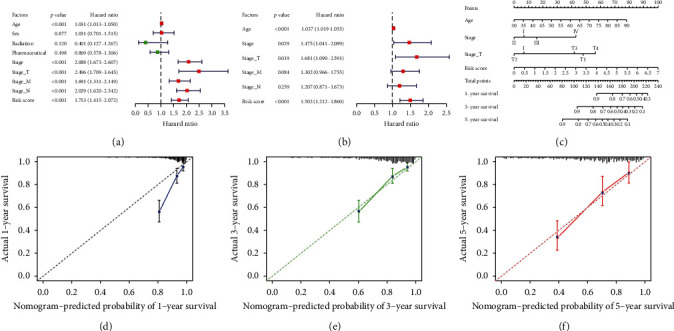
Prognostic value of the TNF family-based signature (TFS) for predicting 1-, 3-, and 5-year OS for CRC patients in the TCGA cohort. (a, b) The univariate and multivariate Cox regression analyses of the TFS and clinical factors. (c) Nomogram for predicting the 1-, 3- and 5-year OS based on the eight-gene TNF. (d–f) Calibration curves of the ability of the nomogram to predict 1-, 3- and 5-year OS.

To evaluate whether the TFS can independently predict CRC prognosis, traditional clinically important factors and the eight-gene TFS were subjected to the univariate and multivariate Cox regression analyses. Age, tumor, node, metastasis (TNM) stage, and the TFS were significantly associated with worse OS based on the univariate Cox regression (Figure 7(a)). The subsequent multivariate Cox regression revealed that both the TNM stage and the TFS were independent prognostic factors in CRC patients in the TCGA cohort (Figure 7(b)). To provide a quantitative tool to predict the prognosis of CRC patients in clinical practice, a nomogram that integrated the eight-gene TFS and TNM stage was constructed (Figure 7(c)). Additionally, the calibration curves for the nomogram showed that it had favorable efficacy for predicting the 1-, 3-, and 5-year OS of patients (Figures 7(d)–7(f)).

## 4. Discussion

As far as we know, this is the first time that a TFS has been constructed to predict prognostic and immunotherapy responses in CRC patients. Using the univariate Cox proportional hazards regression analysis and the LASSO-Cox proportional hazards regression analysis, an eight-gene TFS for CRC was identified, and it was validated using GEO data. The TFS was found to be an independent risk factor for poor OS in patients with CRC. We also investigated the immune profile in high- and low-risk patients and found that the TFS was closely associated with various tumor-infiltrating immune cells. Additionally, the TFS was negatively associated with several immunotherapy response biomarkers, including PD-L1, PD-1, CTLA4, TIGIT, and TIM-3. This indicates that tumor immune escape may contribute to the adverse prognosis of high-risk CRC patients.

The gene expression profiles of TNF family members in CRC patients were systematically analyzed. As a result, eight genes (TNFRSF10B, LTBR, TNFRSF10C, TNFSF11, TNFRSF19, NGFR, TNFRSF11A, and TNFRSF25) were used to establish the TFS. TNFRSF10B (also known as DR5 or TRAILR2) is a protein that belongs to the TNFRSF family and mediates the extrinsic apoptotic pathway in various cancer cells [27]. Recent studies revealed that upregulation of this protein in human CRC cells enhanced the efficiency of cancer therapy [28–30]. The lymphotoxin-beta receptor (LT*β*R or LTBR) is a member of the TNFRSF family, and it may be involved in the promotion of cell proliferation in CRC [31]. TNFRSF10C belongs to the TNFRSF family and can bind to TNF-related apoptosis-inducing ligand-like (TRAIL) to inhibit the intracellular signaling pathway of apoptosis. Additionally, downregulation of this protein aggravates distant CRC metastasis [32]. TNFRSF19 (also known as TROY) is a member of the TNFRSF family that is upregulated in primary CRC, which results in the occurrence or progression of CRC [33]. NGFR belongs to the TNFRSF family and has been shown to be directly or indirectly involved in CRC development and metastasis [34]. Although the prognostic value of TNFSF25, TNFRSF11A, and TNFRSF11 has not been previously investigated in CRC, they still have the potential to be used as novel biomarkers.

Recently, tumor-infiltrating immune cells in the TME have received increased attention due to their important roles in the regulation of cancer progression and predicting cancer outcomes [35]. In this study, immune cell infiltration analyses were performed to compare the inflammatory status between the low- and high-risk groups. We found that the high-risk CRC patients had higher proportions of Treg cells, nonactivated macrophages (M0), and NK cells. Treg cells are a subtype of CD4+ T cell that are critical to the maintenance of immune homeostasis and are involved in tumor immune escape, thereby contributing to tumor development and progression [36]. Similarly, tumor-associated macrophages are an important component of the TME and play a role in tumorigenesis and progression by promoting immune escape [37]. Although NK cells are considered to be major effector cells in both innate immunity and tumor immunosurveillance [38], high infiltration of NK cells has been associated with poor prognosis in some tumors, which may be because tumor-associated macrophages, monocytes, and other immune cells impair their function [39, 40].

Additionally, we investigated the associations between the TFS-based risk subgroups and several immunotherapy response biomarkers, comprising immune checkpoint proteins and TIDE scores. As expected, the high-risk CRC patients generally had lower expression of PD-L1, PD-1, CTLA4, TIGIT, and TIM-3. The results indicate that high-risk patients may have a poor response to ICB [41]. TIDE scores serve as an effective alternative to traditional single biomarkers for predicting ICB responses. A higher TIDE Score not only indicates that the tumor has immune escape phenotypes, but it also predicts a poor response to ICB among cancer patients [21]. Additionally, increased values for IFNG, MSI Score, and CD274 indicate a good response to ICB [42]. We showed that the CRC patients in the high-risk group had positive values regarding TIDE Score, T cell dysfunction, and T cell exclusion, and negative values regarding IFNG, MSI Score, and CD274. Taken together, these results indicate that the poor prognosis of high-risk CRC patients is due to tumor immune evasion and poor response to ICB, which contributes to tumor invasion and metastasis.

The immunoscore summarizes the density of CD3+ and CD8+ T cell effectors at the invasive margin and the core of the tumor [43]. It has been reported to be superior to the TNM classification for predicting OS in CRC [44]. Previous studies reported that CRC patients with a high immunoscore had the lowest risk of recurrence and better OS than those with a low immunoscore [43, 45]. Accordingly, our results showed that patients in the high-risk group had a low immunoscore, which further validate the reliability of the TFS.

However, the study had several limitations that should be noted. First, the data in this study were from the TCGA and GEO databases, and the results should be further clinically validated to evaluate the robustness of the TFS in predicting the prognosis of CRC patients in clinical settings. Second, the predictive ability of the TFS in patients with various clinical characteristics, such as high-fat diet, alcohol consumption, anxiety, and depression was not evaluated. However, these factors contribute to CRC initiation and progression and the differential prognoses of patients [46, 47]. Third, as there were no gene expression data from patients receiving immunotherapy, prospective studies are needed to confirm the ability of the TFS to predict immunotherapy responses.

In summary, this was the first study to identify and validate a reliable, clinically feasible TFS for CRC patients, which has independent predictive value regarding clinical outcomes and immunotherapy responses among these patients.

## Figures and Tables

**Table 1:** Clinical characteristics of the patients from multiple institutions.

Characteristics	TCGA-CRC (*n* = 516)	GSE17536 (*n* = 177)	GSE17537 (*n* = 55)	GSE39582 (*n* = 585)	GSE87211 (*n* = 363)
*Age* (years) (mean)	31-90 (66.18)	26-92 (65.48)	23-94 (62.31)	22-97 (66.95)	35.7-81.5 (62.90)
*Gender*					
Male	235	81	29	263	115
Female	281	96	26	322	248
*Stage*					
I + II	291	81	19	309	NA
III + IV	225	96	36	270	NA
NA				2	NA
0				4	
*OS state*					
Alive	411	104	35	385	304
Dead	105	73	20	194	49
NA				6	10
OS time (months) (mean)	0.03-150.07 (28.15)	0.92-142.55 (48.12)	0.43-111.48 (45.23)	0-201 (57.83)	0-152.48 (54.75)

**Table 2:** The univariate Cox analysis of TNF family genes in the TCGA cohort.

Gene symbol	HR	Aliases	Family	HR.95L	HR.95H	*P* value
CD27	0.9709	TNFRSF7	TNFRSF	0.8151	1.1565	0.7406
CD40	1.0949	TNFRSF5	TNFRSF	0.9181	1.3056	0.3130
CD40LG	1.0877	TNFSF5, CD154	TNFSF	0.7844	1.5083	0.6142
CD70	1.0377	TNFSF7, CD27L	TNFSF	0.8573	1.2560	0.7044
EDA	0.9661	EDA-A1, EDA-A2	TNFSF	0.7994	1.1675	0.7208
EDA2R	0.9405	TNFRSF27, XEDAR	TNFRSF	0.7224	1.2243	0.6484
EDAR	1.0668	EDA-A1R	TNFRSF	0.9437	1.2060	0.3010
FAS	0.8419	TNFRSF6, CD95	TNFRSF	0.6874	1.0311	0.0962
FASLG	0.9453	TNFSF6, CD95-L	TNFSF	0.6999	1.2766	0.7135
LTA	0.9417	TNFSF1	TNFSF	0.6381	1.3896	0.7621
LTB	1.0080	TNFSF3	TNFSF	0.8389	1.2112	0.9322
LTBR	1.4905	TNFRSF3	TNFRSF	0.9610	2.3117	0.0747
NGFR	1.1845	TNFRSF16, CD271	TNFRSF	0.9744	1.4399	0.0891
RELT	1.0834	TNFRSF19L	TNFRSF	0.7789	1.5068	0.6343
TNF	0.8812	TNFSF2, TNFA	TNFSF	0.6434	1.2069	0.4305
TNFRSF10A	0.8024	TRAILR1, CD261	TNFRSF	0.6140	1.0488	0.1071
TNFRSF10B	0.7799	TRAILR2, CD262	TNFRSF	0.5935	1.0248	0.0744
TNFRSF10C	0.7389	TRAILR3, CD263	TNFRSF	0.5563	0.9814	0.0367
TNFRSF10D	0.9766	TRAILR4, CD264	TNFRSF	0.7621	1.2514	0.8515
TNFRSF11A	0.7898	RANK, CD265	TNFRSF	0.6530	0.9554	0.0151
TNFRSF11B	0.9560	OPG	TNFRSF	0.8222	1.1115	0.5582
TNFRSF12A	1.0208	FN14, TWEAKR, CD266	TNFRSF	0.8196	1.2715	0.8542
TNFRSF13B	0.9264	TACI, TNFRSF14B, CD267	TNFRSF	0.5875	1.4606	0.7420
TNFRSF13C	1.1481	BAFFR, CD268	TNFRSF	0.8683	1.5180	0.3326
TNFRSF14	1.3445	LIGHTR, HVEM, CD270	TNFRSF	0.9390	1.9252	0.1061
TNFRSF17	0.8806	BCMA, TNFRSF13A, CD269	TNFRSF	0.7463	1.0390	0.1318
TNFRSF18	1.0049	GITR, AITR, CD357	TNFRSF	0.8154	1.2386	0.9631
TNFRSF19	1.1917	TROY, TAJ	TNFRSF	1.0162	1.3976	0.0309
TNFRSF1A	1.1784	TNFR1, CD120A	TNFRSF	0.7865	1.7654	0.4262
TNFRSF1B	0.8724	TNFR2, CD120B	TNFRSF	0.6804	1.1186	0.2819
TNFRSF21	1.1121	DR6, CD358	TNFRSF	0.7778	1.5902	0.5602
TNFRSF25	1.3221	DR3, TNFRSF12	TNFRSF	1.0482	1.6675	0.0184
TNFRSF4	1.0445	OX40, CD134	TNFRSF	0.8318	1.3116	0.7078
TNFRSF6B	NA	DCR3	TNFRSF	NA	NA	NA
TNFRSF8	0.9705	CD30	TNFRSF	0.6332	1.4876	0.8908
TNFRSF9	0.8403	4-1BB, CD137, ILA	TNFRSF	0.6078	1.1616	0.2922
TNFSF10	0.9383	TRAIL, CD253	TNFSF	0.7388	1.1917	0.6018
TNFSF11	0.8209	RANKL, CD254	TNFSF	0.6601	1.0207	0.0758
TNFSF12	1.1672	TWEAK	TNFSF	0.9247	1.4732	0.1931
TNFSF13	1.0137	APRIL, CD256	TNFSF	0.8102	1.2683	0.9054
TNFSF13B	1.0583	BAFF, CD257	TNFSF	0.8784	1.2750	0.5510
TNFSF14	0.8514	LIGHT, HVEML, CD258	TNFSF	0.5787	1.2526	0.4141
TNFSF15	0.9451	TL1A	TNFSF	0.7111	1.2563	0.6976
TNFSF18	0.8249	GITRL	TNFSF	0.5191	1.3108	0.4152
TNFSF4	0.9764	OX40L, CD134L, CD252	TNFSF	0.7877	1.2104	0.8278
TNFSF8	1.1121	CD30L, CD153	TNFSF	0.7984	1.5490	0.5299
TNFSF9	0.9408	4-1BB-L, CD137L	TNFSF	0.8274	1.0698	0.3521

## Data Availability

Publicly available datasets were analyzed in this study. This data can be found here: TCGA data, https://portal.gdc.cancer.gov/; GEO data (GSE17536, GSE39582, GSE17537, and GSE87211), https://www.ncbi.nlm.nih.gov/geo/.
